# Educational inequalities in TV viewing among older adults: a mediation analysis of ecological factors

**DOI:** 10.1186/1479-5868-10-138

**Published:** 2013-12-19

**Authors:** Katrien De Cocker, Ilse De Bourdeaudhuij, Megan Teychenne, Sarah McNaughton, Jo Salmon

**Affiliations:** 1 Department of Movement and Sports Sciences, Ghent University, Watersportlaan 2, 9000 Gent, Belgium; 2 Research Foundation Flanders (FWO), Egmontstraat 5, 1000 Brussel, Belgium; 3 Centre for Physical Activity and Nutrition Research, School of Exercise and Nutrition Sciences, Deakin University, 221 Burwood Highway, VIC 3125 Burwood, Australia

**Keywords:** Older adults, Education, Sitting, Cohort study

## Abstract

**Background:**

Television (TV) viewing, a prevalent leisure-time sedentary behaviour independently related to negative health outcomes, appears to be higher in less educated and older adults. In order to tackle the social inequalities, evidence is needed about the underlying mechanisms of the association between education and TV viewing. The present purpose was to examine the potential mediating role of personal, social and physical environmental factors in the relationship between education and TV viewing among Australian 55–65 year-old adults.

**Methods:**

In 2010, self-reported data was collected among 4082 adults (47.6% men) across urban and rural areas of Victoria, for the Wellbeing, Eating and Exercise for a Long Life (WELL) study. The mediating role of personal (body mass index [BMI], quality of life), social (social support from family and friends, social participation at proximal level, and interpersonal trust, social cohesion, personal safety at distal level) and physical environmental (neighbourhood aesthetics, neighbourhood physical activity environment, number of televisions) factors in the association between education and TV viewing time was examined using the product-of-coefficients test of MacKinnon based on multilevel linear regression analyses (conducted in 2012).

**Results:**

Multiple mediating analyses showed that BMI (p ≤ 0.01), personal safety (p < 0.001), neighbourhood aesthetics (p ≤ 0.01) and number of televisions (p ≤ 0.01) partly explained the educational inequalities in older adult’s TV viewing. No proximal social factors mediated the education-TV viewing association.

**Conclusions:**

Interventions aimed to reduce TV viewing should focus on personal (BMI) and environmental (personal safety, neighbourhood aesthetics, number of televisions) factors, in order to overcome educational inequalities in sedentary behaviour among older adults.

## Background

Recent studies have revealed that sedentary behaviours are important independent risk factors for all-cause and cardiovascular mortality [1,2], as well as for several other chronic diseases, such as diabetes, cancer, obesity, hypertension, bone/joint-disease, and depression [3,4]. It has been recommended that adults should reduce total sitting time and avoid longer periods of sitting, even when health-related physical activity recommendations are met [4,5]. Population studies using objective and self-reported measurements indicate that the prevalence of sitting time is high in adults [6,7]. Television (TV) viewing, also associated with various deleterious health outcomes [8,9] is one of the most common leisure-time sedentary behaviours among adults in developed countries [10,11]. In Australia for example, adults spend on average 2.4 hours (SD = 1.4) per day watching TV [12].

Several studies, including a recent review [13], have shown that TV viewing is related to socio-economic variables, suggesting that less educated adults are more likely to watch TV compared to adults with a higher level of education [13-17]. This may place less educated people at greater risk of premature morbidity and mortality [13-17]. Prolonged TV viewing time is also associated with older age [12,15]. As the disease burden attributable to chronic disease increases considerably from age 45 onwards [18], older adults are an important target group for health promotion and research. In addition, the majority of health problems associated with older age can be prevented or delayed primarily by lifestyle changes implemented in the 55–65 year age group [18]. In order to overcome educational differences in TV viewing among this older age group, information is needed about the factors influencing this relationship.

One framework used to explain behaviours in public health is the ecological model, which posits that behaviour is influenced by a wide range of factors at multiple levels, such as intrapersonal, interpersonal/social and physical environmental correlates [19]. An ecological model of the correlates of sedentary behaviour was recently described, highlighting the importance of understanding the behaviour setting or context in which sitting occurs [20]. A small body of research has examined ecological factors that may be associated with TV viewing. In Australia, adults reporting high levels of TV viewing were more likely to be female, over 60 years, in the low or inactive physical activity categories, overweight or obese, and with no paid employment [12,15]. Enjoyment of TV, perceived barriers to physical activity, living in rural areas, and low levels of objectively assessed neighbourhood walkability have also been shown to be associated with higher levels of TV viewing in Australia [15,21,22]. Some evidence on the correlates of TV viewing is also available from other continents [23-25]. In summary, socio-demographic (education, age, employment, neighbourhood) and behavioural (weight status, physical activity level) characteristics were the most important identified correlates of TV viewing time.

The correlates of the ecological model might also play a role in explaining socio-economic differences in TV viewing. One study conducted in Australia investigated the contribution of intrapersonal, social and environmental factors to mediating socio-economic (educational) inequalities in women’s TV viewing [17]. It was found that intrapersonal and social factors partly mediated this relationship. However, given that this is currently the only study that has examined the underlying mechanisms explaining the association between education and TV viewing, more research is needed in this area. In addition, the previously mentioned study was in adult (18–65 year) women only [17]. It is possible that different factors may be mediators in different (age) groups. Therefore, the objective of the present study was to investigate the mediating role of ecological factors on the relationship between education and TV viewing in older men and women aged 55–65 years.

## Methods

### Study design, sampling, procedures and participants

This study used the baseline data from the Wellbeing, Eating and Exercise for a Long Life (WELL) study in Australian older adults. In this prospective cohort study of adults aged 55–65 years at baseline, baseline data were collected in 2010 [26,27]. The sample for the WELL study was drawn from the Australian Electoral Commission electoral roll. A total of 84 urban and rural Victorian neighbourhoods of low, medium and high socioeconomic position [classified according to the socioeconomic Index for Areas score (SEIFA)] were selected. Within each of the 84 areas, 134 participants were randomly selected, resulting in a total sampling pool of 11,256.

Between February and April 2010, a sample of adults aged 55–65 years was invited to complete a self-administered postal questionnaire covering nutrition, physical activity, sedentary behaviour, obesity and quality of life. Of the 11,256 postal surveys, 380 were returned as undeliverable and 95 were returned from individuals outside the targeted age range. In total, 4082 adults completed the questionnaire (38% response rate). Participants’ characteristics are presented in Table 1. All participants provided written consent and study protocols were approved by the Deakin University Human Research Ethics Committee (EC2009-105). Further details about the sampling and procedures are published elsewhere [26,27].

**Table 1:** Characteristics of participants

	**All (n=4082)**
**Highest qualification**	
No formal qualifications: n (%)	572 (14.3)^a^
Year 10 or equivalent: n (%)	912 (22.8)^a^
Year 12 or equivalent: n (%)	477 (11.9)^a^
Trade apprentice: n (%)	318 (7.9)^a^
Certificate/diploma: n (%)	641 (16.0)^a^
University degree: n (%)	593 (14.8)^a^
Higher university degree: n (%)	492 (12.3)^a^
*Missing values: n=77*	
**TV viewing time**	
Mean hours/day (SD)	3.33 (2.44)
*Missing values: n=164*	
**Body mass index**	
Mean kg/m² (SD)	27.1 (4.8)
*Missing values: n=169*	
**Quality of life**	
Mean score (SD)	76.9 (16.8)
*Missing values: n=1*	
**Age**	
Mean years (SD)	60.2 (3.2)
*Missing values: n=19*	
**Gender**	
Women: n (%)	2138 (52.4)^a^
Men: n (%)	1944 (47.6)^a^
*Missing values: n=0*	
**Country of birth**	
Australia: n (%)	3252 (80.2)^a^
United Kingdom: n (%)	261 (6.4)^a^
Italy: n (%)	58 (1.4)^a^
Greece: n (%)	26 (0.6)^a^
New Zealand: n (%)	47 (1.2)^a^
Vietnam: n (%)	20 (0.5)^a^
Other: n (%)	384 (9.5)^a^
*Missing values: n=28*	
**Relationship status**	
In no relationship: n (%)	903 (22.3)^a^
In a relationship: n (%)	3149 (77.7)^a^
*Missing values: n=30*	
**Number of children**	
None: n (%)	565 (13.9)^a^
One: n (%)	361 (8.9)^a^
Two: n (%)	1491 (36.8)^a^
Three: n (%)	1024 (25.3)^a^
Four or more: n (%)	613 (15.1)^a^
*Missing values: n=28*	
**Residence**	
Urban: n (%)	1925 (47.2)^a^
Rural: n (%)	2157 (52.8)^a^
*Missing values: n=0*	
**Retirement status**	
Not retired: n (%)	2669 (66.7)^a^
Retired: n (%)	1335 (33.3)^a^
*Missing values: n=78*	
**Smoking status**	
Ever smoked: n (%)	2033 (50.3)^a^
Used to smoke: n (%)	1518 (37.6)^a^
Smokes occasionally: n (%)	138 (3.4)^a^
Smokes regularly: n (%)	351 (8.7)^a^
*Missing values: n=42*	
**Total physical activity**	
Mean minutes/day (SD)	55.1 (33.5)
*Missing values: n=163*	

^a^Percentage values are expressed in relation to the total valid sample (4082 minus missing values).

### Measures

#### Outcome measure: TV viewing time

Participants were asked to estimate the number of hours and minutes they spent sitting watching TV on a weekday and weekend day during the last seven days [21]. This measure showed moderate validity (compared with 3-day behavioural log; ρ = 0.30) [21], which is comparable with validity estimates of similar self-reported measurement methods [11]; and good reliability (ICC = 0.82) in a sample of Australian adults [21].

#### Predictor measure: education

Highest educational level was used as an indicator of individual socio-economic position. This score includes ‘no formal qualifications’, ‘year 10 or equivalent’ (school certificate), ‘year 12 or equivalent’ (high school certificate), trade/apprenticeship’ (e.g. hairdresser, chef), ‘certificate/diploma’ (e.g. childcare, technician), ‘university degree’ or ‘higher university degree’ (e.g. graduate diploma, masters, PhD).

#### Potential mediators: ecological factors

The selection of potential mediators was based on the ecological model of sedentary behaviour describing personal, social and physical environmental factors (see Table 2) that influence sedentary behaviour [20]. A brief description of the potential mediator variables follows and these are described in more detail in Table 2.

**Table 2:** Questionnaire items and response options for the potential mediators

	**Reference**
**Personal factors**	
**BMI:** open-ended questions	
‘What is your weight?’	
‘What is your height?’	
**Quality of life:** score on 100 using SF-36 with assessment on	[28-30]
- Physical functioning	
- Role-physical (role limitations due to physical health)	
- Bodily pain	
- General health	
- Vitality	
- Social functioning	
- Role-emotional (role limitations due to emotional problems)	
- General mental health	
**Social environmental factors**	
***Proximal factors*:**	
**Social support to limit sitting from family:** 5-point scale ranging from ‘never’ to ‘very often’	[31]
‘During the past year, how often did members of your family or people you live with (including spouse/partner) discourage you from sitting around too much (e.g. watching too much TV)?’	
**Social support to limit sitting from friends or work colleagues:** 5-point scale ranging from ‘never’ to ‘very often’	[31]
‘During the past year, how often did friends or work colleagues discourage you from sitting around too much (e.g. watching too much TV)?’	
**Social participation:** not at all / < once per month / 1-2 per month / >2 per month	[32]
Informal social participation (visit to/from family, friends, neighbours)	
Social participation in public spaces (café/restaurant, social club, cinema/theatre, party/dance)	
Social participation in group activities (played sport, attend gym/exercise class, another class, hobby group, singing/acting/music group, self-help or support group)	
***Distal factors*:**	
**Interpersonal trust:** 5-point scale ranging from ‘strongly disagree’ to ‘strongly agree’	[33]
‘Most people can be trusted’	
‘Most of the time people try to be helpful’	
**Social cohesion:** 5-point scale ranging from ‘strongly disagree’ to ‘strongly agree’	[34]
‘People in this neighbourhood can be trusted’	
‘This is a close-knit neighbourhood’	
‘People around here are willing to help their neighbours’	
‘People in this neighbourhood generally don’t get along with each other’ (reverse scored)	
‘People in this neighbourhood do not share the same values’ (reverse scored)	
**Personal safety:** 5-point scale ranging from ‘strongly disagree’ to ‘strongly agree’	[35]
‘I feel safe walking in my neighbourhood, day or night’	
‘Violence is not a problem in my neighbourhood’	
‘My neighbourhood is safe from crime’	
**Physical environmental factors**	
**Neighbourhood aesthetics:** 5-point scale ranging from ‘strongly disagree’ to ‘strongly agree’	[32]
‘There is a lot of rubbish on the street in my neighbourhood’ (reverse scored)	
‘There is a lot of noise in my neighbourhood’ (reverse scored)	
‘In my neighbourhood the buildings and homes are well-maintained’	
‘The buildings and homes in my neighbourhood are interesting’	
‘My neighbourhood is attractive’	
**Neighbourhood physical activity environment:** 5-point scale ranging from ‘strongly disagree’ to ‘strongly agree’	[35]
‘My neighbourhood offers many opportunities to be physically active’	
‘Local sports clubs and other facilities in my neighbourhood offer many opportunities to get exercise’	
‘It is pleasant to walk in my neighbourhood’	
‘The trees in my neighbourhood provide enough shade’	
‘In my neighbourhood it is easy to walk places’	
‘I often see other people walking in my neighbourhood’	
‘I often see other people exercising (e.g. jogging, bicycling, playing sports) in my neighbourhood’	
**Number of televisions in the house:** none / one / two / three / four or more	
‘How many televisions do you have in your house?’	

### Personal factors

Body Mass Index (*BMI;* calculated from self-reported weight and height: weight/height^2^) and *quality of life* assessed using the Medical Outcomes Study Short-Form General Health Survey (SF-36) [28-30] were examined as personal factors.

### Social environmental factors

Three proximal factors (*social support to limit sitting from family*[31], *social support to limit sitting from friends or work colleagues*[31], and *social participation*[32]) and three distal factors (*interpersonal trust*[33], s*ocial cohesion*[34], and *personal safety*[35]) were assessed.

### Physical environmental factors

Perception of the participants’ neighbourhood factors (*neighbourhood aesthetics*[35], *neighbourhood physical activity environment*[35]), and *the number of televisions in the house* were examined.

### Covariates

Age, gender, country of birth, relationship status, number of children, retirement status, region of residence, smoking status, and total physical activity (see categories in Table 1) may influence the outcome variable [12,13]. For that reason, they were tested as potentially confounding factors. As only age (positive correlation) and retirement status (retired people reported higher levels of TV viewing) were found to be associated with TV viewing time, only these variables were included in the analyses as covariates.

### Data reduction and statistical analyses

Cronbach’s alpha coefficients (α) of internal consistency for the potential mediating variables scales were acceptable to good (α=0.68-0.69), except for interpersonal trust (α=0.57). The different items of all potential mediators were summed and scored on the original 5-point scales. Skewed variables (TV viewing time; social support to limit sitting from family or people living with the individual; social support to limit sitting from friends or work colleagues; physical activity) were log-transformed to obtain normal distributions. Clustering at suburb level (n = 84 areas) was taken into account by conducting multi-level analyses. All analyses were adjusted for age and retirement status. An alpha level of p < 0.05 was used to determine statistical significance. Analyses were conducted in 2012, using SPSS for Windows version 19.0 (SPSS Inc., Chicago, IL, USA).

In the first stage of the analyses, the main association between education and TV viewing time was tested (τ-coefficient). To test associations, linear mixed-models analyses were used. In the second stage, the mediating role of the ecological factors on the association between education and TV viewing time was tested using the product-of-coefficients test of MacKinnon [36]. This test consists of different stages: (1) the *action theory tests* which estimate the association between education and the potential mediators (α-coefficients); (2) the *conceptual theory tests* which estimate the association between the potential mediators and the outcome (β-coefficients); (3) the calculation of the product of the two coefficients (αβ), representing the *mediating effect*; and (4) the calculation of dividing αβ by its standard error (SE) to assess the statistical significance of the mediating role (t-value). For the calculation of the SE, the Sobel test was used [SE_αβ_ = √(α^2^ SE_β_^2^ + β^2^ SE_α_^2^)] [37]. The obtained value of αβ/SE_αβ_ was then compared to a standard normal distribution to report on the magnitude of p [36]. If the t value is >1.960, >2.576, or >3.291 there is a significant mediation effect at the 5% level, 1% level, and 0.1% level respectively. Furthermore, the proportion mediating the association between education and TV viewing time was estimated by dividing the product of coefficients (αβ) by the τ-coefficient. Finally, after assessing single mediating models, multiple mediating models were assessed including only those factors that were found to be significant in single mediating models. The total multiple mediation role was examined through a model including all significant single mediators simultaneously. In addition, the independent mediating roles of the mediators in the multiple mediation model were examined through separate models.

## Results

### Study sample

In total, 4082 adults with a mean age of 60.2 (3.2) years participated in this study. About 52% were female, 63% were overweight or obese, and nearly 27% were higher educated (university degree or higher). Additional participant characteristics are reported in Table 1.

### TV viewing time according to education

Overall, participants reported 3.33 (SD = 2.44) hours of TV viewing per day. Those with no formal qualifications reported the highest level of TV viewing time [4.12 (SD = 2.87) hours/day], while those with a higher university degree reported the lowest level [2.36 (SD = 1.74) hours/day]. Mean TV viewing time (hours/day) for the other education groups was 3.63 (SD = 2.61) hours/day for those with only a school certificate; 3.52 (SD = 2.67) hours/day for those with a high school certificate; 3.39 (SD = 2.15) hours/day for with a trade/apprenticeship qualification; 3.12 (SD = 2.23) hours/day for those having a certificate or diploma; and 2.69 (SD = 1.77) hours/day for those with a university degree.

### Main association, action theory and conceptual theory tests

The first stage of the analyses showed an inverse association between education and TV viewing time (see *main association test* in Table 3), indicating that individuals with a higher education, reported lower levels of TV viewing time: per level of education, mean TV viewing time changes by a factor of 1.10 (i.e. back transformation of the log-transformed beta = 0.04), which is 6 minutes for every hour. The associations between education and the separate potential mediators are also presented in Table 3 (see *action theory tests*). Higher levels of education were significantly associated with higher scores for quality of life, social participation, interpersonal trust, social cohesion, personal safety, neighbourhood aesthetics, and neighbourhood physical activity environment. Higher levels of education were significantly associated with lower BMI and less televisions in the house. As a result, only these nine ecological factors were included as potential mediators in the following single-mediator models. No association was found between education and social support to limit sitting.

**Table 3:** **Main association test, action theory tests and conceptual theory tests**^**a**^

**Main association test: association between education and TV viewing time**^**a**^		
	**τ (SE)**	**95% CI**
	-0.04 (0.01)	-0.05, -0.03
**Action theory tests: association between education and potential mediators**^**a**^		
**Potential mediators**	**α (SE)**	**95% CI**
**Personal factors**		
BMI	-0.24 (0.04)	-0.32, -0.16
Quality of life	1.06 (0.13)	0.80, 1.32
**Social environmental factors**		
Support family	-3.06E^-5^(0.00)	0.00, 0.00
Support friends/colleagues	-0.01 (0.00)	-0.01, 0.00
Social participation	0.03 (0.00)	0.03, 0.04
Interpersonal trust	0.04 (0.01)	0.03, 0.05
Social cohesion	0.02 (0.01)	0.01, 0.03
Personal safety	0.04 (0.01)	0.03, 0.06
**Physical environmental factors**		
Neighbourhood aesthetics	0.03 (0.00)	0.02, 0.04
Physical activity environment	0.03 (0.01)	0.02, 0.04
Number of televisions in house	-0.03 (0.01)	-0.04, -0.01
**Conceptual theory tests: association between potential mediators and TV viewing time**^**a**^		
**Potential mediators**	**β (SE)**	**95% CI**
**Personal factors**		
BMI	0.01 (0.00)	0.01, 0.01
Quality of life	0.00 (0.00)	0.00, 0.00
**Social environmental factors**		
Social participation	-0.02 (0.02)	-0.06, 0.02
Interpersonal trust	-0.01 (0.02)	-0.04, 0.03
Social cohesion	-0.01 (0.02)	-0.05, 0.02
Personal safety	-0.05 (0.01)	-0.07, -0.02
**Physical environmental factors**		
Neighbourhood aesthetics	-0.06 (0.02)	-0.09, -0.02
Physical activity environment	-0.05 (0.02)	-0.08, -0.02
Number of televisions in house	0.05 (0.01)	0.03, 0.07

^a^Adjusted for age and retirement status and clustering by neighbourhood area.

*CI* confidence interval.

The potential mediators were then included individually as additional predictors in the models that examined the association between education and TV viewing time. A higher BMI, higher quality of life, lower perceptions of personal safety, neighbourhood aesthetics, neighbourhood physical activity environment and having more televisions in the house were associated with a higher level of TV viewing time (see *conceptual theory tests* in Table 3).

### Mediation role

Results of the single-mediator models are presented in Table 4 (see *single mediation models*). BMI, personal safety, neighbourhood aesthetics, neighbourhood physical activity environment and the number of televisions in the house had significant mediating roles on the association between education and TV viewing time. Quality of life, social participation, interpersonal trust, and social cohesion did not mediate this relationship.

**Table 4:** **Mediating role of socio-ecological factors on association between education and TV viewing**^**a**^

**Mediators**	**αβ (SE)**	**95% CI**	**t (p)**	**% mediation**
**Single mediation models**				
**Personal factors**				
BMI	-0.002 (0.001)	-0.004, -0.001	-3.6***	5.7
Quality of life	-0.002 (0.001)	-0.004, 0.000	-1.9	5.1
**Social environmental factors**				
Social participation	-0.001 (0.001)	-0.002, 0.001	-0.9	1.5
Interpersonal trust	-0.000 (0.001)	-0.002, 0.001	-0.4	0.6
Social cohesion	-0.000 (0.001)	-0.001, 0.000	-1.0	0.5
Personal safety	-0.002 (0.001)	-0.003, -0.001	-6.6***	5.0
**Physical environmental factors**				
Aesthetics	-0.002 (0.001)	-0.002, -0.001	-7.4***	3.7
Physical activity environment	-0.001 (0.001)	-0.002, -0.001	-2.7**	3.3
Number of televisions in house	-0.001 (0.000)	-0.002, -0.001	-3.1**	3.2
**Multiple mediation model**				
**Total mediation role**				
All significant single mediators together	-0.006 (0.002)	-0.009, -0.002	-3.2**	13.4
**Independent mediating role of the mediators in the multiple mediation model**				
BMI	-0.002 (0.001)	-0.003, -0.001	-3.2**	4.5
Personal safety	-0.001 (0.000)	-0.002, -0.001	-4.3***	3.1
Neighbourhood aesthetics	-0.001 (0.000)	-0.001, -0.001	-3.2**	1.5
Physical activity environment	-0.001 (0.001)	-0.002, 0.001	-1.0	1.2
Number of televisions in house	-0.001 (0.000)	-0.002, -0.001	-3.2**	3.0

^a^Adjusted for age and retirement status and clustering by neighbourhood area.

*CI* confidence interval.

**p < 0.01, ***p ≤ 0.001.

Results of the multiple-mediator model including all significant single mediators simultaneously and the independent mediation role of each variable resulting from the multiple-mediation model are presented in Table 4 (see *multiple mediation model*). The proportion mediated by the combination of significant single mediators was 13.4%. BMI, personal safety, neighbourhood aesthetics and the number of televisions in the house had a unique contribution to the explanation of the relationship between education and TV viewing time, and mediated this relation for 4.5%, 3.1%, 1.5% and 3.0% respectively.

## Discussion

The present study aimed to explore the mechanisms which may explain the educational inequalities in TV viewing among Australian older adults. Education was inversely associated with TV viewing, confirming results shown in previous studies [14-17]. The current results showed that these educational inequalities in TV viewing were partly mediated by certain ecological factors, namely BMI, personal safety, neighbourhood aesthetics, and the number of televisions in the house.

BMI had the highest proportion (4.5%) of mediation (which was still relatively low), suggesting that less educated older adults are likely to have higher BMI scores, which in turn may result in higher levels of TV viewing. However, reverse causality between BMI and TV viewing is also possible: low educated individuals who watch more TV, might be at risk of higher BMI scores. In an Australian sample of adult women aged 18–65 years, weight status was also found to be a significant mediator of the association between education and TV viewing [17]. The present finding might suggest that future programs focusing on weight management in less educated older adults might help reduce educational inequalities in TV viewing. However, longitudinal studies are needed to be able to ascertain the direction of the effect.

A second group of factors that were found to be partial mediators (similarly representing relatively low percentages) were the environmental factors. Less educated older adults had lower scores for perceptions of the social (*personal safety*) and physical (*neighbourhood aesthetics*) environment consistent with other studies [38,39], which was also related with higher levels of TV viewing. Less educated adults may be more likely to live in deprived neighbourhoods. As such neighbourhoods may be (perceived as) unsafe and unattractive [40], time spent outdoors might be replaced by sedentary indoor activities, including TV viewing. However, longitudinal studies are needed to confirm the direction of relationships and exclude the possibility that those who watch more TV have less positive perceptions about their neighbourhood. Present findings suggest that targeting perceived environmental variables in less educated older adults might reduce the educational differences in TV viewing. For example, feelings about safety, violence and crime can be addressed in less educated older adults, together with attractiveness (e.g. rubbish, noise) of neighbourhoods. In the present study, proximal social factors (e.g. social participation) did not contribute to explaining the association between education and TV viewing. The normative nature of TV viewing might be one explanation for this as older adults (and their proximal social environment) are possibly not aware of the health consequences of TV viewing. Including an educational-based component to interventions aimed at reducing sedentary behaviour may be useful in order to increase awareness and knowledge of sedentary behaviours, such as TV viewing. Only one other study has assessed the mediating role of ecological factors in the association between education and TV viewing [17]. In contrast to our findings, that study suggested to focus on intrapersonal (enjoyment of TV viewing and weight status) and social (social cohesion and social support from friends for physical activity) strategies. Physical environmental factors were not important in mediating the relationship between education and TV viewing in 18–65 year-old Australian women [17]. The discrepancies in findings between the studies are most likely resulting from the different samples studied. This highlights the need for more research investigating the mediating role of ecological factors on the relationship between education and TV viewing, conducted in various study groups.

The *number of televisions* in the house (physical environmental factor) was likely to be higher in less educated older adults, also explaining higher levels of TV viewing. The fact that less educated individuals had more televisions might seem unexpected, as lower educated individuals are more likely to have a lower income and consequently less resources to buy televisions [41]. However, lower educated adults often buy technological media devices to feel themselves part of the society [42]. Considering the findings of the current study, future interventions might focus on the discouragement of multiple indoor entertainment devices and products, such as TV’s, DVD’s etc. and encourage physical activity equipment at home. The number of televisions in the house was also found to be a significant mediator of the association between maternal education and children’s TV viewing [43]. However, no comparable studies in adults were found.

In summary, the current findings suggest that targeting weight status and (perceived) environmental factors may be a useful approach for limiting TV viewing among older adults with a lower education. Perceptions of the neighbourhood environment may not reflect reality in older adults. Therefore, potential future intervention strategies might include community initiatives to improve personal safety (e.g. by organizing neighbourhood watch) and aesthetics in the neighbourhood.

Limitations of the present study should be acknowledged. Firstly, the data is self-reported which may result in recall and social desirability bias [44]. However, measures were derived from published sources with acceptable validity and reliability levels [21,28,32-35]. Including the use of more objective measures, such as accelerometers or inclinometers, to assess sedentary behaviour, as well as objective measures of the environment [e.g. GIS (Geographic Information System) data] may be an important component of future studies. Although perceptions of the environment are valuable as they impact on behaviour, Ball and colleagues found that there may be relatively poor agreement between perceived and objective measures of physical activity environments [45]. Secondly, the cross-sectional nature of the design does not permit causality of the investigated relationships to be examined. Thirdly, the modest response rate should be taken into account, however there was variation in the demographic and physical activity characteristics of participants. Finally, the list of potential mediators was limited to the present factors tested, and therefore the mediating role of other potential factors (e.g. psychosocial constructs such as self-efficacy, cognitive variables such as intention, other individual factors such as functional status, or other sedentary activities such as computer use) [13] needs to be examined. These factors may differ according to education and might also be associated with TV viewing.

A major strength of this study is that it is the first to examine the underlying mechanisms in the association between education and TV viewing among older adults. Further, the large, random sample of older adults living in both rural and urban neighbourhoods was adequately powered to detect associations after controlling for a number of covariates. Finally, the inclusion of measures that assessed physical and social environmental factors specifically linked to sedentary behaviour was a strength of this study since little previous research has utilised such measures.

## Conclusions

The present results are useful for informing the development of public health strategies and intervention programs to reduce time spent sitting while watching TV among older, less educated adults. The association between education and TV viewing in Australian older adults is partly mediated by personal (BMI), social (personal safety) and physical (neighbourhood aesthetics, number of televisions in the house) environmental factors. Consequently, interventions aimed to reduce TV viewing should focus on these ecological factors, in order to reduce educational inequalities in sedentary behaviour among older adults.

## Abbreviations

TV: Television; WELL: Wellbeing, Eating and Exercise for a Long Life study; SEIFA: Socioeconomic Index for Areas score; BMI: Body mass index; GIS: Geographic Information System.

## Competing interests

The authors declare that they have no competing interests. All authors have no financial disclosures.

## Authors’ contributions

KDC contributed to the conception and design of the study, performed the statistical analysis, interpreted the data, and led the writing of the paper. IDB, MT, SM, JS participated in the conceptualization of the study, helped to interpret the data and provided substantive feedback on the manuscript. All authors have read and approved the final manuscript.
